# Supplementary material for: No magic bullet: Limiting in-school transmission in the face of variable SARS-CoV-2 viral loads
Source: Front Public Health. 2022 Dec 1;10:941773. doi: 10.3389/fpubh.2022.941773 (PMC9751474; doi:10.3389/fpubh.2022.941773)
Supplement: Supplementary file 1 [file Data_Sheet_1.pdf]

Supplementary Materials for “No magic bullet: limiting in-school transmission in the face of variable SARS-CoV-2 viral loads”

Supplementary Text

S1: Children’s susceptibility and infectiousness

Early in the pandemic, there was a perception that children had reduced susceptibility to infection by SARS-CoV-2, based on studies conducted while children had lower contact rates than the general population (1,2). Rigorous meta-analyses do not support children’s reduced susceptibility to infection at this point (1). Consistent with this, studies with surveillance testing strategies report similar rates of infection between school-aged children and adults. For instance, in the UK (October 2020), the highest prevalence of SARS-CoV-2 infections in areas with open schools was in 18-25 year-olds, followed by 11-18 year-olds, with 5-11 year-olds having comparable prevalence to working-age adults (3). At present, within the United States, the American Academy of Pediatrics (4) estimates that greater than 1 in 10 children in the country has tested positive, and children comprised 19.0% of the cumulative reported COVID-19 cases (22.2% of the US population is under the age of 18).

The efficiency with which children transmit SARS-CoV-2 infections has also been debated- a key finding reported and cited often in the early debate about school reopening was that children were not usually the index case (first infection) within in a family (5), suggesting that children may not be responsible for disease spread. However, this finding is confounded with the lower likelihood of detection of asymptomatic index cases, and a number of studies (many of which rely on surveillance testing) point to children’s infectivity being similar (6–10) or even higher (11,12) than that of adults. Notably, similar infectivity between children and adults has been reported for variants of concern such as Alpha (13).

S2: Methodological issues with inferences regarding lack of transmission in school settings

The CDC’s science brief on the topic of in-school SARS-CoV-2 spread states that “the majority of cases that are acquired in the community and are brought into a school setting result in limited

spread inside schools when multiple layered prevention strategies are in place" (14). This inference is based on two metrics.

The first metric is that rates of infection in schools and communities usually track each other closely, as has been reported in numerous studies worldwide. This correlation has been used inappropriately by the CDC and others (15,16) to infer a lack of a causal relationship. In prior work (17), we examined the validity of this inference by simulating a scenario where schools have a higher rate of SARS-CoV-2 transmission than the surrounding community and comparing it to a scenario where the rate of transmission was similar between schools and the community. We found that the ratio of cases between schools and community tracked closely even when schools were driving spread. This finding suggests that the correlation between caseloads in schools and communities is just as likely to be a result of in-school spread driving transmission within communities (cannot use correlation in case counts to infer a lack of causality). In prior work, we have demonstrated that transmission chains originating within schools are capable of generating large chains of spread within the community that can remain undetected in the absence of widespread surveillance testing (18).

The second metric supporting the idea of limited spread in a school setting is that chains of transmission that can be clearly linked to in-school disease spread are rare. However, absence of evidence is not evidence of absence. Symptom-gated forward contact tracing (the method commonly used by schools in the US) to detect child-to-child transmission relies on the appearance and reporting of two consecutive symptomatic cases, connected by a transmission event (for example, see (16)). Because the majority of transmission comes from a minority of cases (overdispersion) (19) and children are more likely to experience asymptomatic infections than adults (20), symptom-gated forward contact tracing is expected to detect only 4.4% of all child-to-child transmission events in schools (17).

In fact, this may point to a problem with symptom-gated forward contact tracing in general-recent contact-tracing studies in the United States suggest that many named contacts are not successfully traced (21,22) and not all symptomatic contacts are willing to undergo testing (23). Consistent with this, other events which may plausibly have led to rampant disease spread were not shown to have done so by symptom-gated forward contact tracing. As an example, consider

the case of the Sturgis motorcycle rally in August 2020, a 10-day event in Meade County, South Dakota attended by approximately 460,000 persons without any mask-wearing requirements or other mitigating policies. The event was followed by a wave of COVID-19 cases in Meade County and South Dakota in the month following the rally, and counties outside of South Dakota that contributed the highest inflows of rally attendees experienced a 6.4-12.5% increase in COVID-19 cases relative to counties without inflows (24). Despite clear evidence of population-level changes in COVID-19 case counts in the weeks following the rally, the CDC and Minnesota Department of Health were able to identify only 21 person-to-person transmission events (25). The methodology used for contact tracing was again, voluntary symptom-gated contact tracing. Out of the 86 positive cases, only 41 reported being in close contact (defined as being within 6 feet of another person for ≥15 minutes) with other people, and they reported an average of 2.5 close contacts. Both statistics are implausible for a 10-day motorcycle rally featuring indoor dining and concerts (26–28). The CDC's report does not specify how many of the 102 secondary contacts were tested- this is also typical for contact-tracing studies in the United States (21,22).

Taken together, this suggests that the rarity of transmission chains in a given setting should be interpreted with caution if the methodology of contact tracing is not transparent, and if voluntary symptom-gated contact tracing methods are used.

S3: Evidence supporting the modeling assumption of aerosol spread of SARS-CoV-2

At this point, a robust body of evidence supports the assumption that SARS-CoV-2 spread occurs primarily through aerosol transmission.

First, efficient indoor transmission is more consistent with aerosol spread than it is with other modes of spread (such as ballistic droplets or surface transmission). In this context, the transmission rate for SARS-CoV-2 has been reported to be many (~19-40) times higher indoors than it is outdoors (29,30). There is also direct evidence for long-range transmission indoors (31,32), even in cases where people were in adjacent rooms (33) or in rooms separated by a

corridor(34) despite never have been in each other's presence. Modeling suggests that long-range transmission may also occur outdoors (35).

Second, infectious virus has been isolated from a number of locations that are consistent with aerosol spread. A number of groups have reported direct isolation of infectious virus from the air (36–38), in air filters and building ducts (39), as well as in exhaled aerosols (40).

Third, epidemiological characteristics of SARS-CoV-2 are consistent with aerosol transmission. For example, aerosol spread provides a direct mechanistic basis for superspreader transmission (41), which has been well documented for SARS-CoV-2 (19). Asymptomatic transmission from people who are not coughing or sneezing – another extensively documented feature of SARS-CoV-2 - is also consistent with aerosol transmission (42,43).

There are a number of excellent overviews on the topic of aerosol spread of SARS-CoV-2 (44,45). The data supporting aerosol transmission of SARS-CoV-2 was also put forth in an open letter to the WHO (46), which the WHO initially contested in a scientific brief (47) before grudgingly accepting (48). It is worth noting that the propensity for aerosol transmission seems to be impacted by evolution as well- for example, the Alpha variant of SARS-CoV-2 has been experimentally shown to be better able to spread via aerosol transmission than the ancestral strain (49).

S4: Evidence supporting the modeling assumption of the room as a well-mixed container

In this work we started with the well-mixed assumption for air flow. This initial assumption is justified from two different lines of evidence.

Physics-based considerations: The current state of evidence suggests a very strong contribution of aerosol particles to the spread of SARS-CoV-2 (summarized in the preceding section). In the context of this work, we have considered particles to be aerosolized if their diameters are in the range of 0.1-30 microns in diameter. For particles in this size range, the rate of spread due to diffusion or Brownian motion is negligible relative to the rate of spread due to air flow. The spread of such particles emitted by an unmasked individual due to speaking (50), coughing or

sneezing (51) would be primarily expected to occur due to the horizontal momentum of exhaled air particles, with a minimal contribution from gravity-induced drift (52). However, in the presence of masks, the horizontal movement of exhaled air is greatly suppressed, and instead the particles rise due to turbulent buoyant airflow (53). The Reynolds number (*Re*) determines the behavior of air flow with a given speed and length scale, with moderately high *Re*s (>100) associated with vortex shedding, and still higher *Re*s (>2000) associated with turbulent flow. In the presence of forced convection within a room (such as can be expected from air circulation or ventilation), *Re* can be expected to be fairly high (≈2000), corresponding to a mix of vortex shedding and turbulent flows (54). Additional factors can also contribute to turbulence, such as human movement.

Epidemiological considerations: A number of epidemiological studies also support the well-mixed-container assumption. Indoor transmission has been demonstrated to occur in a wide variety of settings at ranges that were likely greater than 6 feet apart (31,32,55). A notable example of this was the Skagit Valley Chorale superspreader event, where a weekly rehearsal with 61 masked attendees led to 53 infections– a model-based analysis of this event has argued that the outcome supports the well-mixed assumption empirically (42). Also consistent with the well-mixed assumption is the finding that transmission occurs 19 times more efficiently indoors (29).

The well-mixed assumption is implemented in our modeling using the Wells-Riley approach, first proposed by Wells (56) in 1955 and extended by Riley (57) in 1978. This approach has been found to be broadly applicable for indoor air transmission for other infectious respiratory diseases (58), and has been used to model SARS-CoV-2 transmission by a number of other groups (59).

S5: Estimates for variation in viral load between viral variants and individuals infected with SARS-CoV-2

In this study, we have used the Delta variant as an example of a SARS-CoV-2 viral strain with increased viral load, to exemplify the impact of viral load on guidelines for preventing

transmission. The increased transmission rate of the Delta strain relative to prior SARS-CoV-2 variants has been at least partly attributed to a higher viral load in the nasopharynx. Studies have found RT-qPCR cycle threshold values for Delta that are approximately 6-1000 times higher than prior variants. We note that there is a range of estimates for relative viral load for the Delta variant compared to other variants (see table below).

Wide person-to-person variation in exhaled viral load have also been reported for SARS-CoV-2 infections based on intrinsic factors as well as on the specific activity being undertaken (60).

**Relative viral load of Delta**	**Variant compared**	**Reference**
6.06 times greater	Alpha	(61)
11.48 times greater	Pre-Alpha	(62)
1260 times greater	Pre-Alpha	(63)

Incubation times also vary between different SARS-CoV-2 variants. The incubation period for the original ancestral strain is around 5 days (64,65) while the Delta incubation is shorter (4.3 days (64)). The Omicron variant has an even shorter incubation period, estimated at approximately 3 days (66).

S6: Parameters governing the efficacy of individual control measures

<u>Air filtration:</u>

High-efficiency particulate air (HEPA) filters are defined by the US Environmental Protection Agency to remove at least 99.97% of airborne particles for all particle sizes (67). These filters can be installed in small mobile or large floor-standing air purifiers, which can ideally exchange 5 to 6 times the air volume in the room per hour (68,69). A standard mobile air purification setup in a classroom has been shown experimentally to reduce airborne viral concentrations by 90% within approximately 30 minutes (69).

<u>Ionizers:</u>

Bipolar air ionizers create an electrostatic charge on airborne particles, causing them to be removed from the air by increasing their aggregation rate and deposition rate on surfaces (70).

Smoke particle studies suggest that ionizers can remove between 80-100% of particles from room air (71–73).

Masks:

Face masks have been shown to lower the risk of SARS-CoV-2 transmission (74) by both reducing the number of virions emitted by infected individuals and reducing the amount of virus inhaled by uninfected individuals who are masked. Cloth and surgical masks have typical filtration efficiencies of around 20-95% for droplets or aerosols, with cloth masks having lower efficiencies (20-75% particles filtered out) for smaller particles sizes (0.3-0.5um) (75). N95 and KN95 respirators are rated to remove 95% or more of 0.3um particles (76).

S7: Estimates for minimum infectious dose for SARS-CoV-2

For the ancestral strain of SARS-CoV-2, estimates of infectious dose have been made by diverse methods including CFD modeling (77) and phylogenetic analysis (78). These estimates point to a small number of infectious particles – 6 (78) to 300 (77) - being sufficient to start an infection. The upper end of this range is similar to the infectious dose for SARS-CoV (79), and an order of magnitude lower than that of influenza (80). Here, we used 500 virions as the minimum infectious dose, which is on the upper end of the published range. Estimates of infectious dose for novel variants such as Delta have been a further order of magnitude lower, with some reports suggesting that fewer than ten viral particles may be sufficient to start an infection (63,81,82).

S8: Performance of vaccines in limiting transmission of SARS-CoV-2

Vaccines against SARS-CoV-2 currently possess high levels of efficacy in preventing severe disease and death from COVID-19 (83) and form a critical last line of defense in the public-health strategy. Vaccinal efficacy against severe disease appears somewhat stable over time, which some have suggested is linked to T-cell activity (84).

The impact of vaccines in limiting infection and transmission appears to be dependent on humoral immunity (85), and this impact appears more limited and time-dependent. Estimating

the impact of SARS-CoV-2 vaccines on transmission (VE_t) is challenging, because many vaccine trials did not directly assess vaccine efficacy against transmission, focusing instead on symptomatic infections. As a substantial portion of SARS-CoV-2 infected patients are infectious while asymptomatic (86) or presymptomatic (87) and this proportion rises with breakthrough infections (88–90), estimates for vaccinal efficacy against transmission are biased when considering only symptomatic infections in the denominator (91,92). Despite this overestimation bias for VE_t, recent reports point to a very low degree of vaccinal efficacy against symptomatic disease in some circumstances (93). This suggests that VE_t is even lower and may in some settings be negligible.

Humoral immunity wanes shortly after vaccination, as neutralizing antibodies (nAbs) decline rapidly after the second dose (94,95), with a mean half-life of about four months (96). Consistent with this decline in nAb levels, waning vaccinal immunity against infection has been documented extensively, with substantial loss of protection against infection occurring within the first six months (97,98). While this efficacy against symptomatic infection is restored by booster doses, booster efficacy also declines rapidly (93).

Viral evolution is a second contributing factor to the loss of vaccinal immunity. Viral immune evasion has also been demonstrated to potently reduce the ability of neutralizing antibodies (nAbs) to bind SARS-CoV-2 spike protein - for example, the Omicron variant shows a profound (20-40 fold) reduction in the binding potency of nAbs against the viral spike protein (99–102). Consistent with this, vaccinal efficacy against symptomatic infection with Omicron is severely compromised- for example, for individuals vaccinated with two doses of the Pfizer vaccine, VE against infection is 8.8% (95% CI, 7.0 to 10.5) at 25 or more weeks (93). Other viral variants have also demonstrated substantial reductions in nAb binding potency (103–105) and vaccinal immunity against infection (106,107). A number of studies have pointed to a predictive relationship between nAb binding potency and vaccinal protection against infection (85,96).

In addition, the impact of vaccination on onward transmission by infected individuals has also been found to be modest. Some reports from earlier in the pandemic indicated a 50% reduction in infectiousness associated with vaccine breakthrough cases with the ancestral strain (108). However, the reduction in viral load for breakthrough cases is minimal for recent variants such

as Delta (85,109–111) and Omicron (112). For these variants, epidemiological data is also consistent with a picture of efficient transmission by breakthrough cases- the secondary attack rate for infection resulting from a vaccinal breakthrough case is only marginally lower than that of unvaccinated individuals for Delta (109,110) and Omicron (113). Multiple real-world examples of superspreader events (114) and ongoing spread (115) among highly vaccinated populations add further weight to the inference of limited impact of vaccination on curbing SARS-CoV-2 spread. Going forward, continued viral evolution and waning vaccinal effectiveness (116) in reducing viral load (which has now been noted for the booster dose as well (117,118) can be further expected to impact the vaccinal reduction of transmission.

In summary, these findings support the assumption that vaccination status does not contribute significantly to the ability of an individual to contract or transmit SARS-CoV-2 in a congregate setting.

References for supplemental materials

1. Davies NG, Klepac P, Liu Y, Prem K, Jit M, Eggo RM. Age-dependent effects in the transmission and control of COVID-19 epidemics. *Nat Med* (2020) 26:1205–1211. doi: 10.1038/s41591-020-0962-9

2. Viner RM, Mytton OT, Bonell C, Melendez-Torres GJ, Ward J, Hudson L, Waddington C, Thomas J, Russell S, van der Klis F, et al. Susceptibility to SARS-CoV-2 Infection Among Children and Adolescents Compared With Adults: A Systematic Review and Meta-analysis. *JAMA Pediatrics* (2021) 175:143–156. doi: 10.1001/jamapediatrics.2020.4573

3. Flasche S, Edmunds WJ. The role of schools and school-aged children in SARS-CoV-2 transmission. *Lancet Infect Dis* (2021) 21:298–299. doi: 10.1016/S1473-3099(20)30927-0

4. American Academy of Pediatrics. Children and COVID-19: State-Level Data Report. *American Academy of Pediatrics* (2022) http://www.aap.org/en/pages/2019-novel-coronavirus-covid-19-infections/children-and-covid-19-state-level-data-report/ [Accessed March 15, 2022]

5. Zhu Y, Bloxham CJ, Hulme KD, Sinclair JE, Tong ZWM, Steele LE, Noye EC, Lu J, Xia Y, Chew KY, et al. A Meta-analysis on the Role of Children in Severe Acute Respiratory Syndrome Coronavirus 2 in Household Transmission Clusters. *Clin Infect Dis* (2021) 72:e1146–e1153. doi: 10.1093/cid/ciaa1825

6. Lopez AS, Hill M, Antezano J, Vilven D, Rutner T, Bogdanow L, Claflin C, Kracalik IT, Fields VL, Dunn A, et al. Transmission Dynamics of COVID-19 Outbreaks Associated with Child Care Facilities — Salt Lake City, Utah, April–July 2020. *MMWR Morb Mortal Wkly Rep* (2020) 69:1319–1323. doi: 10.15585/mmwr.mm6937e3

7. Laxminarayan R, Wahl B, Dudala SR, Gopal K, Mohan B C, Neelima S, Jawahar Reddy KS, Radhakrishnan J, Lewnard JA. Epidemiology and transmission dynamics of COVID-19 in two Indian states. *Science* (2020) 370:691–697. doi: 10.1126/science.abd7672

8. Grijalva CG, Rolfes MA, Zhu Y, McLean HQ, Hanson KE, Belongia EA, Halasa NB, Kim A, Reed C, Fry AM, et al. Transmission of SARS-COV-2 Infections in Households — Tennessee and Wisconsin, April–September 2020. *MMWR Morb Mortal Wkly Rep* (2020) 69:1631–1634. doi: 10.15585/mmwr.mm6944e1

9. Park YJ, Choe YJ, Park O, Park SY, Kim Y-M, Kim J, Kweon S, Woo Y, Gwack J, Kim SS, et al. Contact Tracing during Coronavirus Disease Outbreak, South Korea, 2020. *Emerging Infectious Diseases* (2020) 26: doi: 10.3201/eid2610.201315

10. Yonker LM, Boucau J, Regan J, Choudhary MC, Burns MD, Young N, Farkas EJ, Davis JP, Moschovis PP, Bernard Kinane T, et al. Virologic Features of Severe Acute Respiratory Syndrome Coronavirus 2 Infection in Children. *The Journal of Infectious Diseases* (2021) 224:1821–1829. doi: 10.1093/infdis/jiab509

11. Paul LA, Daneman N, Schwartz KL, Science M, Brown KA, Whelan M, Chan E, Buchan SA. Association of Age and Pediatric Household Transmission of SARS-CoV-2 Infection. *JAMA Pediatrics* (2021) 175:1151–1158. doi: 10.1001/jamapediatrics.2021.2770

12. Heald-Sargent T, Muller WJ, Zheng X, Rippe J, Patel AB, Kociolek LK. Age-Related Differences in Nasopharyngeal Severe Acute Respiratory Syndrome Coronavirus 2 (SARS-CoV-2) Levels in Patients With Mild to Moderate Coronavirus Disease 2019 (COVID-19). *JAMA Pediatrics* (2020) 174:902–903. doi: 10.1001/jamapediatrics.2020.3651

13. Loenenbach A, Markus I, Lehfeld A-S, an der Heiden M, Haas W, Kiegele M, Ponzi A, Unger-Goldinger B, Weidenauer C, Schlosser H, et al. SARS-CoV-2 variant B.1.1.7 susceptibility and infectiousness of children and adults deduced from investigations of childcare centre outbreaks, Germany, 2021. *Eurosurveillance* (2021) 26: doi: 10.2807/1560-7917.ES.2021.26.21.2100433

14. CDC. Science Brief: Transmission of SARS-CoV-2 in K-12 Schools and Early Care and Education Programs. *Centers for Disease Control and Prevention* (2021) https://www.cdc.gov/coronavirus/2019-ncov/science/science-briefs/transmission_k_12_schools.html [Accessed March 15, 2022]

15. Ertem Z, Schechter-Perkins EM, Oster E, van den Berg P, Epshtein I, Chaiyakunapruk N, Wilson FA, Perencevich E, Pettey WBP, Branch-Elliman W, et al. The impact of school opening model on SARS-CoV-2 community incidence and mortality. *Nat Med* (2021) 27:2120–2126. doi: 10.1038/s41591-021-01563-8

16. Falk A, Benda A, Falk P, Steffen S, Wallace Z, Høeg TB. COVID-19 Cases and Transmission in 17 K-12 Schools - Wood County, Wisconsin, August 31-November 29, 2020. *MMWR Morb Mortal Wkly Rep* (2021) 70:136–140. doi: 10.15585/mmwr.mm7004e3

17. Johnson KE, Lachmann M, Stoddard M, Pasco R, Fox SJ, Meyers LA, Chakravarty A. Detecting in-school transmission of SARS-CoV-2 from case ratios and documented clusters. (2021)2021.04.26.21256136. doi: 10.1101/2021.04.26.21256136

18. Johnson KE, Stoddard M, Nolan RP, White DE, Hochberg NS, Chakravarty A. In the long shadow of our best intentions: Model-based assessment of the consequences of school reopening during the COVID-19 pandemic. *PLoS One* (2021) 16:e0248509. doi: 10.1371/journal.pone.0248509

19. Endo A, Abbott S, Kucharski AJ, Funk S. Estimating the overdispersion in COVID-19 transmission using outbreak sizes outside China. *Wellcome Open Res* (2020) 5:67. doi: 10.12688/wellcomeopenres.15842.3

20. Dawood FS, Porucznik CA, Veguilla V, Stanford JB, Duque J, Rolfes MA, Dixon A, Thind P, Hacker E, Castro MJE, et al. Incidence Rates, Household Infection Risk, and Clinical Characteristics of SARS-CoV-2 Infection Among Children and Adults in Utah and New

York City, New York. *JAMA Pediatrics* (2022) 176:59–67. doi: 10.1001/jamapediatrics.2021.4217

21. McClain C, Rainie L. The Challenges of Contact Tracing as U.S. Battles COVID-19. *Pew Research Center* (2020) https://www.pewresearch.org/internet/2020/10/30/the-challenges-of-contact-tracing-as-u-s-battles-covid-19/ [Accessed March 20, 2022]

22. Hendrix MJ, Walde C, Findley K, Trotman R. Absence of Apparent Transmission of SARS-CoV-2 from Two Stylists After Exposure at a Hair Salon with a Universal Face Covering Policy - Springfield, Missouri, May 2020. *MMWR Morb Mortal Wkly Rep* (2020) 69:930–932. doi: 10.15585/mmwr.mm6928e2

23. Doyle T, Kendrick K, Troelstrup T, Gumke M, Edwards J, Chapman S, Propper R, Rivkees SA, Blackmore C. COVID-19 in Primary and Secondary School Settings During the First Semester of School Reopening — Florida, August–December 2020. *MMWR Morb Mortal Wkly Rep* (2021) 70:437–441. doi: 10.15585/mmwr.mm7012e2

24. Dave D, McNichols D, Sabia JJ. The contagion externality of a superspreading event: The Sturgis Motorcycle Rally and COVID-19. *South Econ J* (2020) doi: 10.1002/soej.12475

25. Firestone MJ, Wienkes H, Garfin J, Wang X, Vilen K, Smith KE, Holzbauer S, Plumb M, Pung K, Medus C, et al. COVID-19 Outbreak Associated with a 10-Day Motorcycle Rally in a Neighboring State — Minnesota, August–September 2020. *MMWR Morb Mortal Wkly Rep* (2020) 69:1771–1776. doi: 10.15585/mmwr.mm6947e1

26. Rapier G. "If I die from the virus, it was just meant to be": 250,000 descend upon tiny South Dakota town for world-famous motorcycle rally. *Business Insider* (2020) https://www.businessinsider.com/sturgis-motorcycle-rally-kicks-off-despite-surging-coronavirus-cases-2020-8 [Accessed March 15, 2022]

27. Associated Press. Harleys everywhere, masks nowhere: Sturgis expects crowd of 250,000. *Chicago Sun-Times* (2020) https://chicago.suntimes.com/coronavirus/2020/8/7/21359318/sturgis-motorcycle-harleys-everywhere-masks-nowhere [Accessed March 15, 2022]

28. 2020 Sturgis Motorcycle Rally attracts thousands with no mask requirements amid pandemic. *USA Today* (2020) https://www.usatoday.com/picture-gallery/news/nation/2020/08/09/2020-sturgis-motorcycle-rally-draws-thousands-no-mask-requirements-covid-19-coronavirus/3331908001/ [Accessed March 15, 2022]

29. Bulfone TC, Malekinejad M, Rutherford GW, Razani N. Outdoor Transmission of SARS-CoV-2 and Other Respiratory Viruses: A Systematic Review. *The Journal of Infectious Diseases* (2021) 223:550–561. doi: 10.1093/infdis/jiaa742

30. Nishiura H, Oshitani H, Kobayashi T, Saito T, Sunagawa T, Matsui T, Wakita T, Team MC-19 R, Suzuki M. Closed environments facilitate secondary transmission of coronavirus disease 2019 (COVID-19). (2020)2020.02.28.20029272. doi: 10.1101/2020.02.28.20029272

31. Ou C, Hu S, Luo K, Yang H, Hang J, Cheng P, Hai Z, Xiao S, Qian H, Xiao S, et al. Insufficient ventilation led to a probable long-range airborne transmission of SARS-CoV-2 on two buses. *Building and Environment* (2022) 207:108414. doi: 10.1016/j.buildenv.2021.108414

32. Lu J, Gu J, Li K, Xu C, Su W, Lai Z, Zhou D, Yu C, Xu B, Yang Z. COVID-19 Outbreak Associated with Air Conditioning in Restaurant, Guangzhou, China, 2020. *Emerg Infect Dis* (2020) 26:1628–1631. doi: 10.3201/eid2607.200764

33. Eichler N, Thornley C, Swadi T, Devine T, McElnay C, Sherwood J, Brunton C, Williamson F, Freeman J, Berger S, et al. Transmission of Severe Acute Respiratory Syndrome Coronavirus 2 during Border Quarantine and Air Travel, New Zealand (Aotearoa). *Emerg Infect Dis* (2021) 27:1274–1278. doi: 10.3201/eid2705.210514

34. Fox-Lewis A, Williamson F, Harrower J, Ren X, Sonder GJB, McNeill A, de Ligt J, Geoghegan JL. Airborne Transmission of SARS-CoV-2 Delta Variant within Tightly Monitored Isolation Facility, New Zealand (Aotearoa). *Emerg Infect Dis* (2022) 28:501–509. doi: 10.3201/eid2803.212318

35. Gorbunov B. Aerosol Particles Generated by Coughing and Sneezing of a SARS-CoV-2 (COVID-19) Host Travel over 30 m Distance. *Aerosol Air Qual Res* (2021) 21:200468. doi: 10.4209/aaqr.200468

36. van Doremalen N, Bushmaker T, Morris DH, Holbrook MG, Gamble A, Williamson BN, Tamin A, Harcourt JL, Thornburg NJ, Gerber SI, et al. Aerosol and Surface Stability of SARS-CoV-2 as Compared with SARS-CoV-1. *New England Journal of Medicine* (2020) 382:1564–1567. doi: 10.1056/NEJMc2004973

37. Lednicky JA, Lauzardo M, Fan ZH, Jutla A, Tilly TB, Gangwar M, Usmani M, Shankar SN, Mohamed K, Eiguren-Fernandez A, et al. Viable SARS-CoV-2 in the air of a hospital room with COVID-19 patients. *International Journal of Infectious Diseases* (2020) 100:476–482. doi: 10.1016/j.ijid.2020.09.025

38. Lednicky JA, Lauzardo M, Alam MM, Elbadry MA, Stephenson CJ, Gibson JC, Morris JG. Isolation of SARS-CoV-2 from the air in a car driven by a COVID patient with mild illness. *International Journal of Infectious Diseases* (2021) 108:212–216. doi: 10.1016/j.ijid.2021.04.063

39. Nissen K, Krambrich J, Akaberi D, Hoffman T, Ling J, Lundkvist Å, Svensson L, Salaneck E. Long-distance airborne dispersal of SARS-CoV-2 in COVID-19 wards. *Sci Rep* (2020) 10:19589. doi: 10.1038/s41598-020-76442-2

40. Adenaiye OO, Lai J, Bueno de Mesquita PJ, Hong F, Youssefi S, German J, Tai S-HS, Albert B, Schanz M, Weston S, et al. Infectious Severe Acute Respiratory Syndrome Coronavirus 2 (SARS-CoV-2) in Exhaled Aerosols and Efficacy of Masks During Early Mild Infection. *Clinical Infectious Diseases* (2021)ciab797. doi: 10.1093/cid/ciab797

41. Lewis D. Superspreading drives the COVID pandemic — and could help to tame it. *Nature* (2021) 590:544–546. doi: 10.1038/d41586-021-00460-x

42. Miller SL, Nazaroff WW, Jimenez JL, Boerstra A, Buonanno G, Dancer SJ, Kurnitski J, Marr LC, Morawska L, Noakes C. Transmission of SARS-CoV-2 by inhalation of respiratory aerosol in the Skagit Valley Chorale superspreading event. *Indoor Air* (2021) 31:314–323. doi: 10.1111/ina.12751

43. Johansson MA, Quandelacy TM, Kada S, Prasad PV, Steele M, Brooks JT, Slayton RB, Biggerstaff M, Butler JC. SARS-CoV-2 Transmission From People Without COVID-19 Symptoms. *JAMA Netw Open* (2021) 4:e2035057. doi: 10.1001/jamanetworkopen.2020.35057

44. Greenhalgh T, Jimenez JL, Prather KA, Tufekci Z, Fisman D, Schooley R. Ten scientific reasons in support of airborne transmission of SARS-CoV-2. *The Lancet* (2021) 397:1603–1605. doi: 10.1016/S0140-6736(21)00869-2

45. Nazaroff WW. Indoor aerosol science aspects of SARS-CoV-2 transmission. *Indoor Air* (2022) 32:e12970. doi: 10.1111/ina.12970

46. Morawska L, Milton DK. It Is Time to Address Airborne Transmission of Coronavirus Disease 2019 (COVID-19). *Clinical Infectious Diseases* (2020) 71:2311–2313. doi: 10.1093/cid/ciaa939

47. WHO. Transmission of SARS-CoV-2: implications for infection prevention precautions. *World Health Organization* (2020) https://www.who.int/news-room/commentaries/detail/transmission-of-sars-cov-2-implications-for-infection-prevention-precautions [Accessed March 15, 2022]

48. WHO. Coronavirus disease (COVID-19): How is it transmitted? *World Health Organization* (2021) https://www.who.int/news-room/questions-and-answers/item/coronavirus-disease-covid-19-how-is-it-transmitted [Accessed March 15, 2022]

49. Port JR, Yinda CK, Avanzato VA, Schulz JE, Holbrook MG, van Doremalen N, Shaia C, Fischer RJ, Munster VJ. Increased small particle aerosol transmission of B.1.1.7 compared with SARS-CoV-2 lineage A in vivo. *Nat Microbiol* (2022) 7:213–223. doi: 10.1038/s41564-021-01047-y

50. Abkarian M, Mendez S, Xue N, Yang F, Stone HA. Speech can produce jet-like transport relevant to asymptomatic spreading of virus. *Proc Natl Acad Sci U S A* (2020) 117:25237–25245. doi: 10.1073/pnas.2012156117

51. Bourouiba L, Dehandschoewercker E, Bush JWM. Violent expiratory events: on coughing and sneezing. *Journal of Fluid Mechanics* (2014) 745:537–563. doi: 10.1017/jfm.2014.88

52. Zhdanov VP, Kasemo B. Virions and respiratory droplets in air: Diffusion, drift, and contact with the epithelium. *Biosystems* (2020) 198:104241. doi: 10.1016/j.biosystems.2020.104241

53. Chen W, Zhang N, Wei J, Yen H-L, Li Y. Short-range airborne route dominates exposure of respiratory infection during close contact. *Building and Environment* (2020) 176:106859. doi: 10.1016/j.buildenv.2020.106859

54. Bazant MZ, Bush JWM. A guideline to limit indoor airborne transmission of COVID-19. *Proc Natl Acad Sci U S A* (2021) 118:e2018995118. doi: 10.1073/pnas.2018995118

55. Vernez D, Schwarz S, Sauvain J-J, Petignat C, Suarez G. Probable aerosol transmission of SARS-CoV-2 in a poorly ventilated courtroom. *Indoor Air* (2021) 31:1776–1785. doi: 10.1111/ina.12866

56. Wells WF. *Airborne Contagion and Air Hygiene*. Cambridge, MA: Harvard University Press (1955).

57. Riley EC, Murphy G, Riley RL. Airborne spread of measles in a suburban elementary school. *Am J Epidemiol* (1978) 107:421–432. doi: 10.1093/oxfordjournals.aje.a112560

58. Sze To GN, Chao CYH. Review and comparison between the Wells–Riley and dose-response approaches to risk assessment of infectious respiratory diseases. *Indoor Air* (2010) 20:2–16. doi: 10.1111/j.1600-0668.2009.00621.x

59. Li H, Shankar SN, Witanachchi CT, Lednicky JA, Loeb JC, Alam MM, Fan ZH, Mohamed K, Eiguren-Fernandez A, Wu C-Y. Environmental Surveillance and Transmission Risk Assessments for SARS-CoV-2 in a Fitness Center. *Aerosol Air Qual Res* (2021) 21:210106. doi: 10.4209/aaqr.210106

60. Verma R, Kim E, Degner N, Walter KS, Singh U, Andrews JR. Variation in Severe Acute Respiratory Syndrome Coronavirus 2 Bioaerosol Production in Exhaled Breath. *Open Forum Infectious Diseases* (2022) 9:ofab600. doi: 10.1093/ofid/ofab600

61. Earnest R, Uddin R, Matluk N, Renzette N, Siddle KJ, Loreth C, Adams G, Tomkins-Tinch CH, Petrone ME, Rothman JE, et al. Comparative transmissibility of SARS-CoV-2 variants Delta and Alpha in New England, USA. (2021)2021.10.06.21264641. doi: 10.1101/2021.10.06.21264641

62. Teyssou E, Delagrèverie H, Visseaux B, Lambert-Niclot S, Brichler S, Ferre V, Marot S, Jary A, Todesco E, Schnuriger A, et al. The Delta SARS-CoV-2 variant has a higher viral load than the Beta and the historical variants in nasopharyngeal samples from newly diagnosed COVID-19 patients. *J Infect* (2021) 83:e1–e3. doi: 10.1016/j.jinf.2021.08.027

63. Li B, Deng A, Li K, Hu Y, Li Z, Shi Y, Xiong Q, Liu Z, Guo Q, Zou L, et al. Viral infection and transmission in a large, well-traced outbreak caused by the SARS-CoV-2 Delta variant. *Nat Commun* (2022) 13:460. doi: 10.1038/s41467-022-28089-y

64. Grant R, Charmet T, Schaeffer L, Galmiche S, Madec Y, Platen CV, Chény O, Omar F, David C, Rogoff A, et al. Impact of SARS-CoV-2 Delta variant on incubation, transmission settings and vaccine effectiveness: Results from a nationwide case-control study in France. *The Lancet Regional Health – Europe* (2022) 13: doi: 10.1016/j.lanepe.2021.100278

65. Lauer SA, Grantz KH, Bi Q, Jones FK, Zheng Q, Meredith HR, Azman AS, Reich NG, Lessler J. The Incubation Period of Coronavirus Disease 2019 (COVID-19) From Publicly Reported Confirmed Cases: Estimation and Application. *Ann Intern Med* (2020) 172:577–582. doi: 10.7326/M20-0504

66. Jansen L, Tegomoh B, Lange K, Showalter K, Figliomeni J, Abdalhamid B, Iwen PC, Fauver J, Buss B, Donahue M. Investigation of a SARS-CoV-2 B.1.1.529 (Omicron) Variant Cluster — Nebraska, November–December 2021. *MMWR Morb Mortal Wkly Rep* (2021) 70:1782–1784. doi: 10.15585/mmwr.mm705152e3

67. US EPA. What is a HEPA filter? *United States Environmental Protection Agency* (2019) https://www.epa.gov/indoor-air-quality-iaq/what-hepa-filter-1 [Accessed March 15, 2022]

68. Curtius J, Granzin M, Schrod J. Testing mobile air purifiers in a school classroom: Reducing the airborne transmission risk for SARS-CoV-2. *Aerosol Science and Technology* (2021) 55:586–599. doi: 10.1080/02786826.2021.1877257

69. Duill FF, Schulz F, Jain A, Krieger L, van Wachem B, Beyrau F. The Impact of Large Mobile Air Purifiers on Aerosol Concentration in Classrooms and the Reduction of Airborne Transmission of SARS-CoV-2. *Int J Environ Res Public Health* (2021) 18:11523. doi: 10.3390/ijerph182111523

70. Jiang S-Y, Ma A, Ramachandran S. Negative Air Ions and Their Effects on Human Health and Air Quality Improvement. *Int J Mol Sci* (2018) 19:2966. doi: 10.3390/ijms19102966

71. Uk Lee B, Yermakov M, Grinshpun SA. Removal of fine and ultrafine particles from indoor air environments by the unipolar ion emission. *Atmospheric Environment* (2004) 38:4815–4823. doi: 10.1016/j.atmosenv.2004.06.010

72. Sawant VS, Meena GS, Jadhav DB. Effect of Negative Air Ions on Fog and Smoke. *Aerosol Air Qual Res* (2012) 12:1007–1015. doi: 10.4209/aaqr.2011.11.0214

73. Grinshpun SA, Mainelis G, Trunov M, Adhikari A, Reponen T, Willeke K. Evaluation of ionic air purifiers for reducing aerosol exposure in confined indoor spaces. *Indoor Air* (2005) 15:235–245. doi: 10.1111/j.1600-0668.2005.00364.x

74. Wang X, Ferro EG, Zhou G, Hashimoto D, Bhatt DL. Association Between Universal Masking in a Health Care System and SARS-CoV-2 Positivity Among Health Care Workers. *JAMA* (2020) 324:703–704. doi: 10.1001/jama.2020.12897

75. Li X, Ding P, Deng F, Mao Y, Zhou L, Ding C, Wang Y, Luo Y, Zhou Y, MacIntyre CR, et al. Wearing time and respiratory volume affect the filtration efficiency of masks against

aerosols at different sizes. *Environ Technol Innov* (2022) 25:102165. doi: 10.1016/j.eti.2021.102165

76. National Personal Protective Technology Laboratory. The Respiratory Protection Information Trusted Source. *Centers for Disease Control and Prevention* (2021) https://www.cdc.gov/niosh/npptl/topics/respirators/disp_part/respsource3basic.html [Accessed March 15, 2022]

77. Basu S. Computational characterization of inhaled droplet transport in the upper airway leading to SARS-CoV-2 infection. (2020)2020.07.27.20162362. doi: 10.1101/2020.07.27.20162362

78. Wang D, Wang Y, Sun W, Zhang L, Ji J, Zhang Z, Cheng X, Li Y, Xiao F, Zhu A, et al. Population Bottlenecks and Intra-host Evolution During Human-to-Human Transmission of SARS-CoV-2. *Front Med* (2021) 8: doi: 10.3389/fmed.2021.585358

79. Watanabe T, Bartrand TA, Weir MH, Omura T, Haas CN. Development of a dose-response model for SARS coronavirus. *Risk Anal* (2010) 30:1129–1138. doi: 10.1111/j.1539-6924.2010.01427.x

80. Nikitin N, Petrova E, Trifonova E, Karpova O. Influenza virus aerosols in the air and their infectiousness. *Adv Virol* (2014) 2014:859090. doi: 10.1155/2014/859090

81. Lythgoe KA, Hall M, Ferretti L, Cesare M de, MacIntyre-Cockett G, Trebes A, Andersson M, Otecko N, Wise EL, Moore N, et al. SARS-CoV-2 within-host diversity and transmission. *Science* (2021) doi: 10.1126/science.abg0821

82. Martin MA, Koelle K. Comment on "Genomic epidemiology of superspreading events in Austria reveals mutational dynamics and transmission properties of SARS-CoV-2." *Science Translational Medicine* 13:eabh1803. doi: 10.1126/scitranslmed.abh1803

83. Abu-Raddad LJ, Chemaitelly H, Butt AA. Effectiveness of the BNT162b2 Covid-19 Vaccine against the B.1.1.7 and B.1.351 Variants. *New England Journal of Medicine* (2021) 385:187–189. doi: 10.1056/NEJMc2104974

84. Moss P. The T cell immune response against SARS-CoV-2. *Nat Immunol* (2022) 23:186–193. doi: 10.1038/s41590-021-01122-w

85. Cromer D, Steain M, Reynaldi A, Schlub TE, Wheatley AK, Juno JA, Kent SJ, Triccas JA, Khoury DS, Davenport MP. Neutralising antibody titres as predictors of protection against SARS-CoV-2 variants and the impact of boosting: a meta-analysis. *Lancet Microbe* (2022) 3:e52–e61. doi: 10.1016/S2666-5247(21)00267-6

86. Vella F, Senia P, Ceccarelli M, Vitale E, Maltezou H, Taibi R, Lleshi A, Venanzi Rullo E, Pellicanò GF, Rapisarda V, et al. Transmission mode associated with coronavirus disease 2019: a review. *Eur Rev Med Pharmacol Sci* (2020) 24:7889–7904. doi: 10.26355/eurrev_202007_22296

87. Cheng H-Y, Jian S-W, Liu D-P, Ng T-C, Huang W-T, Lin H-H, Taiwan COVID-19 Outbreak Investigation Team. Contact Tracing Assessment of COVID-19 Transmission Dynamics in Taiwan and Risk at Different Exposure Periods Before and After Symptom Onset. *JAMA Intern Med* (2020) 180:1156–1163. doi: 10.1001/jamainternmed.2020.2020

88. Novazzi F, Taborelli S, Baj A, Focosi D, Maggi F. Asymptomatic SARS-CoV-2 Vaccine Breakthrough Infections in Health Care Workers Identified Through Routine Universal Surveillance Testing. *Ann Intern Med* (2021)M21-3486. doi: 10.7326/M21-3486

89. Rovida F, Cassaniti I, Paolucci S, Percivalle E, Sarasini A, Piralla A, Giardina F, Sammartino JC, Ferrari A, Bergami F, et al. SARS-CoV-2 vaccine breakthrough infections with the alpha variant are asymptomatic or mildly symptomatic among health care workers. *Nat Commun* (2021) 12:6032. doi: 10.1038/s41467-021-26154-6

90. Riemersma KK, Grogan BE, Kita-Yarbro A, Halfmann PJ, Segaloff HE, Kocharian A, Florek KR, Westergaard R, Bateman A, Jeppson GE, et al. Shedding of Infectious SARS-CoV-2 Despite Vaccination. (2021)2021.07.31.21261387. doi: 10.1101/2021.07.31.21261387

91. Williams LR, Ferguson NM, Donnelly CA, Grassly NC. Measuring vaccine efficacy against infection and disease in clinical trials: sources and magnitude of bias in COVID-19 vaccine efficacy estimates. (2021)2021.07.30.21260912. doi: 10.1101/2021.07.30.21260912

92. Lin D-Y, Gu Y, Zeng D, Janes HE, Gilbert PB. Evaluating Vaccine Efficacy Against SARS-CoV-2 Infection. (2021)2021.04.16.21255614. doi: 10.1101/2021.04.16.21255614

93. Andrews N, Stowe J, Kirsebom F, Toffa S, Rickeard T, Gallagher E, Gower C, Kall M, Groves N, O'Connell A-M, et al. Covid-19 Vaccine Effectiveness against the Omicron (B.1.1.529) Variant. *New England Journal of Medicine* (2022) doi: 10.1056/NEJMoa2119451

94. Widge AT, Rouphael NG, Jackson LA, Anderson EJ, Roberts PC, Makhene M, Chappell JD, Denison MR, Stevens LJ, Pruijssers AJ, et al. Durability of Responses after SARS-CoV-2 mRNA-1273 Vaccination. *N Engl J Med* (2021) 384:80–82. doi: 10.1056/NEJMc2032195

95. Notarte KI, Guerrero-Arguero I, Velasco JV, Ver AT, Santos de Oliveira MH, Catahay JA, Khan MSR, Pastrana A, Juszczyk G, Torrelles JB, et al. Characterization of the significant decline in humoral immune response six months post-SARS-CoV-2 mRNA vaccination: A systematic review. *J Med Virol* (2022) doi: 10.1002/jmv.27688

96. Khoury DS, Cromer D, Reynaldi A, Schlub TE, Wheatley AK, Juno JA, Subbarao K, Kent SJ, Triccas JA, Davenport MP. Neutralizing antibody levels are highly predictive of immune protection from symptomatic SARS-CoV-2 infection. *Nat Med* (2021) 27:1205–1211. doi: 10.1038/s41591-021-01377-8

97. Chemaitelly H, Tang P, Hasan MR, AlMukdad S, Yassine HM, Benslimane FM, Al Khatib HA, Coyle P, Ayoub HH, Al Kanaani Z, et al. Waning of BNT162b2 Vaccine Protection against SARS-CoV-2 Infection in Qatar. *New England Journal of Medicine* (2021) 385:e83. doi: 10.1056/NEJMoa2114114

98. Levin EG, Lustig Y, Cohen C, Fluss R, Indenbaum V, Amit S, Doolman R, Asraf K, Mendelson E, Ziv A, et al. Waning Immune Humoral Response to BNT162b2 Covid-19 Vaccine over 6 Months. *New England Journal of Medicine* (2021) 385:e84. doi: 10.1056/NEJMoa2114583

99. Schubert M, Bertoglio F, Steinke S, Heine PA, Ynga-Durand MA, Maass H, Sammartino JC, Cassaniti I, Zuo F, Du L, et al. Human serum from SARS-CoV-2-vaccinated and COVID-19 patients shows reduced binding to the RBD of SARS-CoV-2 Omicron variant. *BMC Med* (2022) 20:102. doi: 10.1186/s12916-022-02312-5

100. Wall EC, Wu M, Harvey R, Kelly G, Warchal S, Sawyer C, Daniels R, Hobson P, Hatipoglu E, Ngai Y, et al. Neutralising antibody activity against SARS-CoV-2 VOCs B.1.617.2 and B.1.351 by BNT162b2 vaccination. *Lancet* (2021) 397:2331–2333. doi: 10.1016/S0140-6736(21)01290-3

101. Cele S, Jackson L, Khoury DS, Khan K, Moyo-Gwete T, Tegally H, San JE, Cromer D, Scheepers C, Amoako DG, et al. Omicron extensively but incompletely escapes Pfizer BNT162b2 neutralization. *Nature* (2022) 602:654–656. doi: 10.1038/s41586-021-04387-1

102. Carreño JM, Alshammary H, Tcheou J, Singh G, Raskin AJ, Kawabata H, Sominsky LA, Clark JJ, Adelsberg DC, Bielak DA, et al. Activity of convalescent and vaccine serum against SARS-CoV-2 Omicron. *Nature* (2022) 602:682–688. doi: 10.1038/s41586-022-04399-5

103. Lucas C, Vogels CBF, Yildirim I, Rothman JE, Lu P, Monteiro V, Gehlhausen JR, Campbell M, Silva J, Tabachnikova A, et al. Impact of circulating SARS-CoV-2 variants on mRNA vaccine-induced immunity. *Nature* (2021) 600:523–529. doi: 10.1038/s41586-021-04085-y

104. Davis C, Logan N, Tyson G, Orton R, Harvey WT, Perkins JS, Mollett G, Blacow RM, COVID-19 Genomics UK (COG-UK) Consortium, Peacock TP, et al. Reduced neutralisation of the Delta (B.1.617.2) SARS-CoV-2 variant of concern following vaccination. *PLoS Pathog* (2021) 17:e1010022. doi: 10.1371/journal.ppat.1010022

105. Garcia-Beltran WF, Lam EC, St Denis K, Nitido AD, Garcia ZH, Hauser BM, Feldman J, Pavlovic MN, Gregory DJ, Poznansky MC, et al. Multiple SARS-CoV-2 variants escape neutralization by vaccine-induced humoral immunity. *Cell* (2021) 184:2372-2383.e9. doi: 10.1016/j.cell.2021.03.013

106. Buchan SA, Chung H, Brown KA, Austin PC, Fell DB, Gubbay JB, Nasreen S, Schwartz KL, Sundaram ME, Tadrous M, et al. Effectiveness of COVID-19 vaccines against Omicron or Delta symptomatic infection and severe outcomes. (2022)2021.12.30.21268565. doi: 10.1101/2021.12.30.21268565

107. Lefèvre B, Tondeur L, Madec Y, Grant R, Lina B, Werf S van der, Rabaud C, Fontanet A. Beta SARS-CoV-2 variant and BNT162b2 vaccine effectiveness in long-term care facilities in France. *The Lancet Healthy Longevity* (2021) 2:e685–e687. doi: 10.1016/S2666-7568(21)00230-0

108. Abu-Raddad LJ, Chemaitelly H, Ayoub HH, Tang P, Coyle P, Hasan MR, Yassine HM, Benslimane FM, Khatib HAA, Kanaani ZA, et al. Effect of vaccination and of prior infection on infectiousness of vaccine breakthrough infections and reinfections. (2021)2021.07.28.21261086. doi: 10.1101/2021.07.28.21261086

109. Acharya CB, Schrom J, Mitchell AM, Coil DA, Marquez C, Rojas S, Wang CY, Liu J, Pilarowski G, Solis L, et al. No Significant Difference in Viral Load Between Vaccinated and Unvaccinated, Asymptomatic and Symptomatic Groups When Infected with SARS-CoV-2 Delta Variant. (2021)2021.09.28.21264262. doi: 10.1101/2021.09.28.21264262

110. Chia PY, Ong SWX, Chiew CJ, Ang LW, Chavatte J-M, Mak T-M, Cui L, Kalimuddin S, Chia WN, Tan CW, et al. Virological and serological kinetics of SARS-CoV-2 Delta variant vaccine breakthrough infections: a multicentre cohort study. *Clinical Microbiology and Infection* (2021) 0: doi: 10.1016/j.cmi.2021.11.010

111. Kissler SM, Fauver JR, Mack C, Tai CG, Breban MI, Watkins AE, Samant RM, Anderson DJ, Metti J, Khullar G, et al. Viral Dynamics of SARS-CoV-2 Variants in Vaccinated and Unvaccinated Persons. *New England Journal of Medicine* (2021) 385:2489–2491. doi: 10.1056/NEJMc2102507

112. Boucau J, Marino C, Regan J, Uddin R, Choudhary MC, Flynn JP, Chen G, Stuckwisch AM, Mathews J, Liew MY, et al. Duration of viable virus shedding in SARS-CoV-2 omicron variant infection. *medRxiv* (2022)2022.03.01.22271582. doi: 10.1101/2022.03.01.22271582

113. Lyngse FP, Mortensen LH, Denwood MJ, Christiansen LE, Møller CH, Skov RL, Spiess K, Fomsgaard A, Lassaunière MM, Rasmussen M, et al. SARS-CoV-2 Omicron VOC Transmission in Danish Households. (2021)2021.12.27.21268278. doi: 10.1101/2021.12.27.21268278

114. Brandal LT, MacDonald E, Veneti L, Ravlo T, Lange H, Naseer U, Feruglio S, Bragstad K, Hungnes O, Ødeskaug LE, et al. Outbreak caused by the SARS-CoV-2 Omicron variant in Norway, November to December 2021. *Eurosurveillance* (2021) 26: doi: 10.2807/1560-7917.ES.2021.26.50.2101147

115. Brown CM, Vostok J, Johnson H, Burns M, Gharpure R, Sami S, Sabo RT, Hall N, Foreman A, Schubert PL, et al. Outbreak of SARS-CoV-2 Infections, Including COVID-19 Vaccine Breakthrough Infections, Associated with Large Public Gatherings — Barnstable County, Massachusetts, July 2021. *MMWR Morb Mortal Wkly Rep* (2021) 70:1059–1062. doi: 10.15585/mmwr.mm7031e2

116. Levine-Tiefenbrun M, Yelin I, Alapi H, Katz R, Herzel E, Kuint J, Chodick G, Gazit S, Patalon T, Kishony R. Viral loads of Delta-variant SARS-CoV-2 breakthrough infections

after vaccination and booster with BNT162b2. *Nat Med* (2021) 27:2108–2110. doi: 10.1038/s41591-021-01575-4

117. Kuhlmann C, Mayer CK, Claassen M, Maponga T, Burgers WA, Keeton R, Riou C, Sutherland AD, Suliman T, Shaw ML, et al. Breakthrough infections with SARS-CoV-2 omicron despite mRNA vaccine booster dose. *Lancet* (2022) 399:625–626. doi: 10.1016/S0140-6736(22)00090-3

118. Levine-Tiefenbrun M, Yelin I, Alapi H, Herzel E, Kuint J, Chodick G, Gazit S, Patalon T, Kishony R. Waning of SARS-CoV-2 booster viral-load reduction effectiveness. *Nat Commun* (2022) 13:1237. doi: 10.1038/s41467-022-28936-y

Supplementary Figures

A

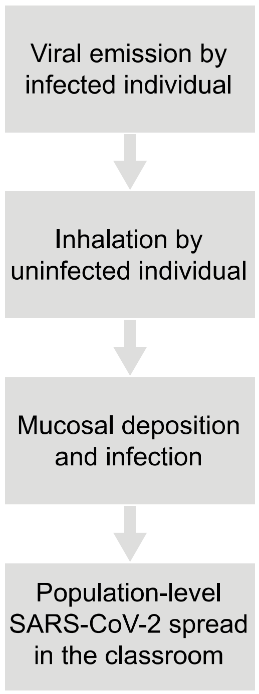

B

Parameter name	Value
Classroom size	4000 cubic feet
Low viral load emission rate	5000 virions/hr
Intermediate viral load emission rate	4,307,619 virions/hr
High viral load emission rate	5,000,000 virions/hr
Viral particle lifetime	1.6 hr
# of virions needed to initiate infection	500
Respiratory tidal volume	0.5 L
Respiratory rate	15 breaths/min

Figure S1: Modeling approach to estimate transmission risk in the classroom. **A:** Model schematic. **B:** Table of parameter values used in differential equations model simulating SARS-CoV-2 emission and inhalation in classrooms.

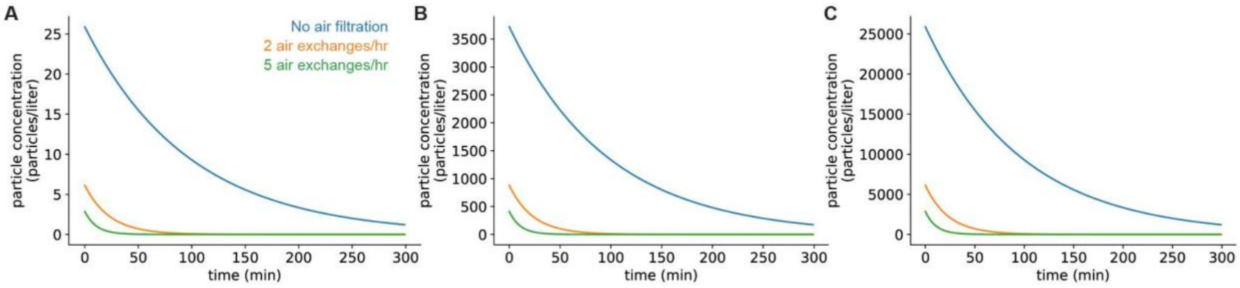

Figure S2: Viral concentrations in a room after the infected individual leaves. The concentration of SARS-CoV-2 in room air is initially at steady state. **A:** Infected individual with the ancestral strain of virus, **B:** Individual infected with the Omicron variant. **C:** Individual infected with the Delta variant. In all panels, the blue curve shows the concentration when there is no ventilation in the room, the orange curve shows the concentration when there is 2 air exchanges/hr, and the green curve shows the concentration when there is 6 air exchanges/hr.

Supplementary Tables

Droplet size (μm)	Percent of expelled droplets	Deposition probability
0.10	0.16	1.34
0.20	0.16	1.60
0.30	0.16	1.53
0.40	0.16	1.51
0.50	0.16	1.47
0.60	0.16	1.47
0.70	0.16	1.52
0.80	0.16	1.52
0.90	0.16	1.48
1.00	0.16	1.43
1.50	0.16	1.74
2.00	0.16	1.98
2.50	0.16	2.49
3.00	0.16	2.82
3.50	0.16	3.70
4.00	0.16	4.77
4.50	0.16	5.61
5.00	2.44	5.97
6.00	2.44	6.71
7.00	2.44	6.09
8.00	2.44	5.36
9.00	2.44	4.69
10.00	1.70	4.07
11.00	1.70	3.54
12.00	1.70	3.09
13.00	1.70	2.94
14.00	1.70	2.73
15.00	0.90	2.18
16.00	0.90	1.55
17.00	0.90	1.23
18.00	0.90	0.78
19.00	0.90	0.73
20.00	0.78	0.52
21.00	0.78	0.41
22.00	0.78	0.29
23.00	0.78	0.17
24.00	0.78	0.07
25.00	0.84	0.01
26.00	0.84	0.00
27.00	0.84	0.01
28.00	0.84	0.00
29.00	0.84	0.00
30.00	0.88	0.00

Table S1: Estimating the fraction of inhaled viruses that are deposited in the nasopharynx based on CFD results.
